# Antibodies against phosphorylcholine in hospitalized versus non-hospitalized obese subjects

**DOI:** 10.1038/s41598-021-99615-z

**Published:** 2021-10-12

**Authors:** Amra Jujić, J. Korduner, H. Holm, G. Engström, E. Bachus, P. Bhattacharya, P. M. Nilsson, Johan Frostegård, M. Magnusson

**Affiliations:** 1 Department of Clinical Sciences, Clinical Research Centre, Lund University, Room 60-12-009, Box 50332, 202 13 Malmö, Sweden; 2 Department of Internal Medicine, Skåne University Hospital, Malmö, Sweden; 3 Department of Cardiology, Skåne University Hospital, Malmö, Sweden; 4 Lund University Diabetes Centre, Lund University, Malmö, Sweden; 5 Institute of Environmental Medicine, Karolinska Institutet, IMM, Nobels väg 13, 17165 Stockholm, Sweden; 6 Wallenberg Center for Molecular Medicine, Lund University, Lund, Sweden; 7 Hypertension in Africa Research Team (HART), North-West University, Potchefstroom, South Africa

**Keywords:** Inflammation, Innate immunity, Biomarkers, Cardiology

## Abstract

Obesity associates with reduced life expectancy, type 2 diabetes, hypertension and cardiovascular disease, and is characterized by chronic inflammation. Phosphorylcholine (PC) is an epitope on oxidized low-density lipoprotein, dead cells and some microorganisms. Antibodies against PC (anti-PC) have anti-inflammatory properties. Here, we explored the role of anti-PC in hospitalized versus non-hospitalized obese. One-hundred-and-twenty-eight obese (BMI ≥ 30 kg/m^2^) individuals (59.8 (± 5.5) years, 53.9% women) from the Malmö Diet and Cancer Cardiovascular Cohort were examined and IgM, IgG1 and IgG2 anti-PC were analyzed by ELISA. Individuals with at least one recorded history of hospitalization prior to study baseline were considered hospitalized obese (HO). Associations between IgM, IgG1 and IgG2 anti-PC and HO (n = 32)/non-hospitalized obese (NHO) (n = 96), but also with metabolic syndrome and diabetes were analysed using logistic regressions. Both IgM and IgG1 anti-PC were inversely associated with HO, also after controlling for age and sex. When further adjusted for waist circumference, systolic blood pressure, glucose levels and smoking status, only IgG1 anti-PC remained significantly associated with HO. In multivariate models, each 1 standard deviation of increment in anti-PC IgG1 levels was inversely associated with prevalence of HO (odds ratio 0.57; CI 95% 0.33–0.98; p = 0.044). IgG2 anti-PC did not show any associations with HO. Low levels of IgM and IgG1 anti-PC are associated with higher risk of being a HO individual independent of sex and age, IgG1 anti-PC also independently of diabetes and metabolic syndrome. The anti-inflammatory properties of these antibodies may be related to inflammation in obesity and its complications.

## Introduction

Obesity is rapidly becoming one of the most alarming public health hazards worldwide, accounting for an increasing negative impact on health due to its deleterious effects of excess body fat accumulation^1^. It is one of the leading risk factors for developing several debilitating comorbidities, such as various atherosclerotic processes (including cardiovascular disease, CVD) and type 2 diabetes^2^. However, although obesity is commonly associated with deleterious metabolic profiles there are individual differences, displaying a heterogeneous phenomenon of obesity. These individuals typically present with a more favorable lipid- and glucometabolic profile along with the absence of other components usually associated with the metabolic syndrome (MetS)^3,4^.

Although insulin resistance is an immensely important risk factor for the development of CVD through the promotion of atherosclerotic processes^5^, other harmful elements may include immunological mechanisms which through inflammatory responses interact with the atherosclerotic plaque, subsequently leading to its rupture and the development of CVD caused by tissue ischemia^6^. Atherosclerotic plaques are characterized by accumulation of oxidized low-density lipoprotein (OxLDL), dead cells and a low-grade inflammation where immune competent cells as T cells, macrophages and dendritic cells represent major contributors. OxLDL is taken up by macrophages which develop into inert foam cells^6^. OxLDL is also pro-inflammatory, and phosphorylcholine (PC), exposed on LDL surface during oxidation, may play a major role, also in OxLDL-induced immune activation. PC is also exposed on dead cells and on some microorganisms, including both bacteria, parasites and nematodes, and is both a danger- and pathogen-associated molecular pattern (DAMP and PAMP)^6^. Antibodies against PC (anti-PC) are present in healthy adults; as much as 5–10% of circulating immunoglobulin M (IgM) consists of IgM anti-PC^6,7^. IgM anti-PC is negatively associated with several chronic inflammatory conditions, including atherosclerosis, CVD, rheumatic diseases and chronic kidney disease (CKD). Potential underlying mechanisms have been described, including anti-inflammatory^6^. As atherosclerosis and is subsequent pro-inflammatory induction is one of the main pathophysiological mechanisms linked to obesity-related mortality and morbidity^8^, one interesting aspect would be to investigate if the levels of anti-PC play a protective role in obesity. Thus, the aim of this observational, cross-sectional study was to determine if anti-PC immunoglobulin M (IgM), G1 (IgG1) and G2 (IgG2) are associated with higher risk of being a hospitalized obese subject.

## Subjects and methods

**Figure 1:**
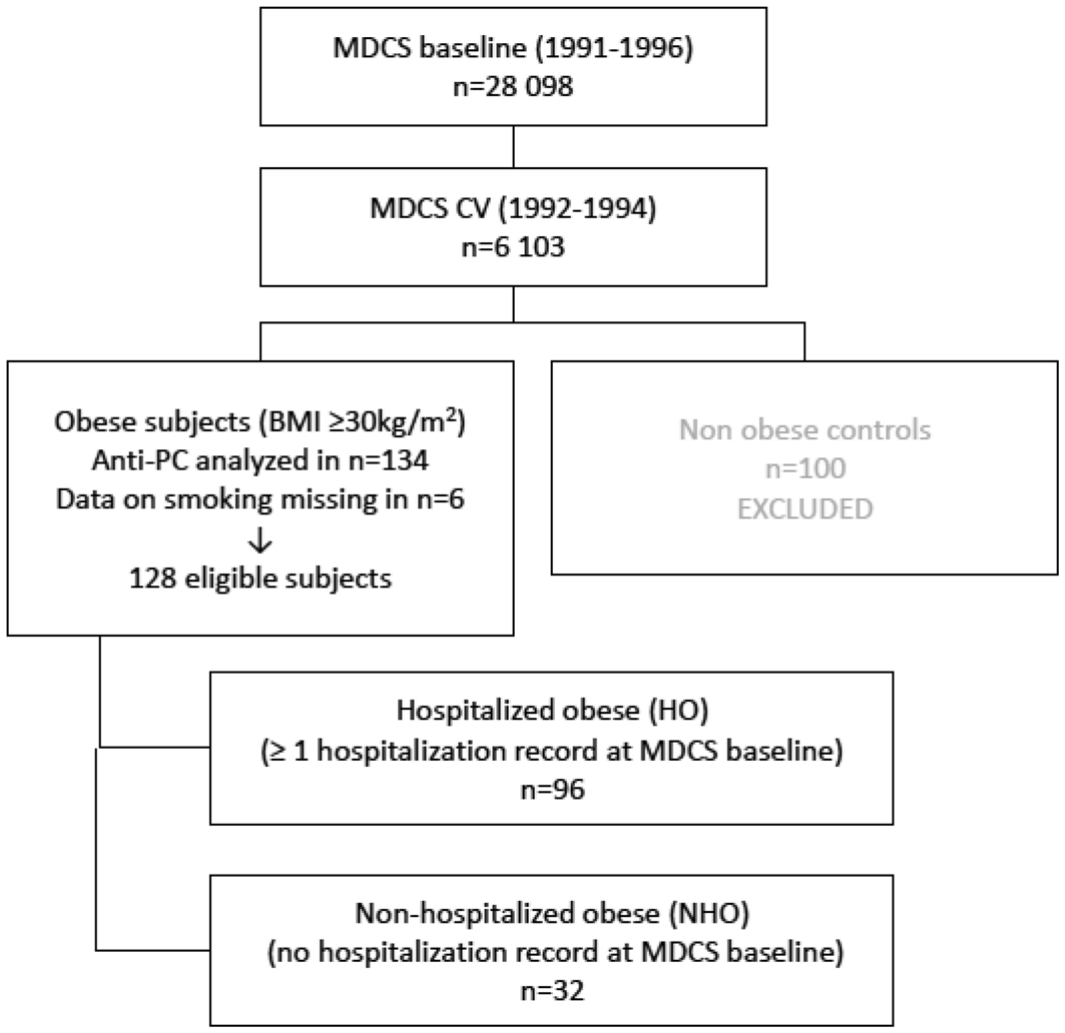
Flow chart of the MDCS-CV sub-cohort stratified for obese and non-obese subjects, respectively.

The Malmö Diet and Cancer Study (MDCS) is a population‐based study that enrolled 28 449 individuals between 1991 and 1996 in the city of Malmö, Sweden. A random sample (every second individual between 1992 and 1994) of the study subjects were invited to participate in a sub-study on the epidemiology of carotid artery disease. This sub-sample comprised the MDCS-Cardiovascular Cohort (MDCS-CV; n = 6103). For this study, the purpose was to randomly select a total of 300 individuals from the MDCS-CV cohort with predefined BMI criteria, data on prior hospitalization status, and equal sex distribution. This resulted in a total of 234 included individuals, due to lack of sufficient number of individuals fulfilling applicable inclusion criteria (see Fig. 1). In a sub-sample of 134 people with obesity (BMI ≥ 30 kg/m^2^) subjects, anti-PC were analyzed. Self-reported data on smoking was missing in six subjects, resulting in 128 subjects with complete data. Those subjects were further sub-divided into two different categories: absence or presence of hospitalization for somatic disease prior to study entrance, as recorded in the Swedish National Hospital Inpatient Register. Hospitalizations due to intoxications/external injuries or normal deliveries were considered non-hospitalizations. People with obesity with no recorded history of hospitalization prior to study entrance (n = 32; 25%) where defined as non-hospitalized obese (NHO). Corresponding individuals with at least one recorded history of hospitalization prior to study entrance (n = 96; 75%) were defined as hospitalized obese (HO)^9^.

The study was approved by the University of Lund research ethics committee (LU 51/90) and is in accordance with the Declaration of Helsinki. All subjects gave informed consent before entering the study. All experiments were performed in accordance with relevant guidelines and regulations.

Anthropometric measurements of weight (kg) and height (cm) were carried out without shoes and in light indoor clothing. Waist circumference (cm) was measured in the standing position without clothing. Right-arm blood pressure (mmHg) was measured twice in the recumbent position after a 5-min rest (Korotkoff phase V). Diabetes was defined as self-reported physician´s diagnosis per questionnaire, or current treatment with anti-diabetic drugs or fasting whole blood glucose ≥ 6.1 mmol/L. Data on medication and smoking status (current smoker yes/no) was retrieved through questionnaires.

### Definitions

General obesity was defined as BMI ≥ 30 kg/m^2^. Abdominal obesity was defined as waist circumference ≥ 88 cm and ≥ 102 cm for women and men, respectively. Metabolic syndrome (MetS) was defined as presence of any three of the following five criteria: abdominal obesity, elevated triglycerides (≥ 1.7 mmol/L), reduced high density lipoprotein (HDL) cholesterol (< 1.03 mmol/L in males and < 1.29 mmol/L in females), increased blood pressure (BP) (systolic ≥ 130 mmHg and/or diastolic ≥ 85 mmHg, or drug treatment), or elevated fasting glucose (≥ 5.6 mmol/L or glucose-lowering treatment)^10^.

### Laboratory assays

Venous blood samples were drawn and stored at − 80 °C until later analysis (2020). Fasting blood glucose (FBG), triglycerides, total cholesterol, LDL and HDL cholesterol were all analyzed at the Department of Clinical Chemistry, Skåne University Hospital, Malmö, participating in a national standardization and quality control system.

### Antibody determinations

Antibodies such as IgM, IgG1 and IgG2 to PC were determined by ELISA essentially as described previously^6,11–13^. Briefly, pooled serum from Sigma Aldrich (St Louis, MO, USA) was used as standard in each plate. Nunc Immuno microwell plates (Thermo Labsystems, Franklin, MA, USA) were coated with PC-bovine serum albumin (BSA) antigen at a concentration of 10 μg/mL per well and incubated overnight at 4 °C. After four washings with wash buffer, the plates were blocked with 2% BSA–phosphate buffered saline (PBS) for 1 h at room temperature. The same washing steps were followed throughout the assay. Serum samples were then diluted at 1:100 for IgM, IgG1 and IgG2 in 0.2% BSA–PBS and added at 100 μL/well to each plate. Plates were then incubated at room temperature for 2 h and washed as described above. Biotin-conjugated goat antihuman IgM, mouse antihuman IgG1 and mouse antihuman IgG2 (diluted 1:30,000, 1:500 and 1:5000 respectively in 1% BSA–PBS) was then added at 100 μL/well and the plates were incubated at room temperature for 2 h. After four washings, horseradish peroxidase conjugated streptavidin (diluted 1:5000, 1:3000 and 1:3000 for IgM, IgG1 and IgG2 respectively in 0.2% BSA–PBS) [Thermo Scientific, Roskilde, Denmark] were added at 100 μL/well to respective plates and they were further incubated for 20 min. The colour was developed by adding the horseradish peroxidase substrate, TMB (3,3′,5,5′ tetramethylbenzidine; Sigma Aldrich, St. Louis, MO, USA), at 100 μL/well and after incubating the plates for 15 min, 20 min and 20 min for IgM, IgG1 and IgG2 respectively at room temperature in a dark place. Further reaction was stopped by adding stop solution 1N H_2_SO_4_ at 50 μL/well to each plate. Finally, plates were read on ELISA Multiscan Plus spectrophotometer (Spectra Max 250; Molecular Devices, CA) at both 450 nm and 540 nm. All samples were measured in duplicates within a single assay and the coefficient of variation between the duplicates was below 15% for all the antibodies.

### Statistics

Variables are presented as means (± standard deviation, SD) or median (25–75 interquartile range, IQR). A stratified random sample was created for identification of eligible subjects for the study. NHO and HO subjects were compared using one-way ANOVA tests for normally distributed continuous variables, Mann–Whitney U test for continuous variables with non-normal distribution, or χ^2^ tests for binary variables. Prior to analyses, variables with non-normal distribution were ln-transformed (anti-PC IgM, IgG1, IgG2, FBG, triglycerides and total cholesterol). Anti-PCs were further z-transformed. Unadjusted logistic regressions were carried out for anti-PC and prevalence of HO using odds ratios (OR) and 95% confidence intervals (95% CI). Multivariate logistic regressions were then carried out adjusted for age and sex (*Model 1*), and further adjusted for waist circumference, systolic blood pressure (SBP), FBG, and smoking status (*Model 2*). All analyses were carried out using SPSS 25.0. A p-value of less than 0.05 was considered significant.

## Results

**Figure 2:**
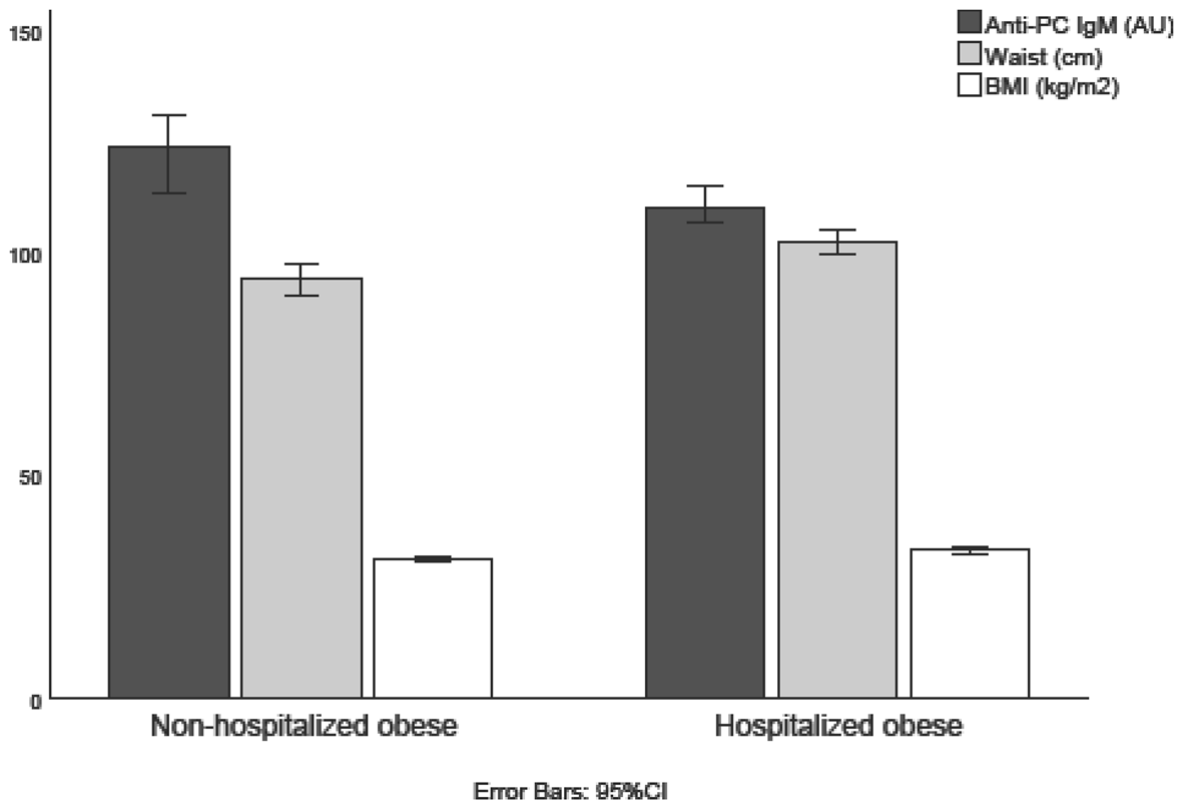
Anti-PC levels, waist circumference and BMI in non-hospitalized obese subjects vs hospitalized obese subjects. Values are median (anti-PC IgM) or mean (waist circumference and BMI). Error bars represent the 95% confidence interval.

Characteristics of the study population are presented in Table 1. HO subjects were older, with higher BMI, waist circumference, SBP and DBP, but lower levels of anti-PC IgM and IgG1 than NHO subjects. Further, a larger proportion of the HO subjects presented with DM and abdominal obesity as compared to subjects with NHO. Anti-PC IgM and IgG1 levels along with waist circumference and BMI in NHO/HO subjects are illustrated in Fig. 2. The 96 HO subjects were hospitalized prior to study entrance for following reasons based on ICD8: Morbi infectiosi ex origine intestinali (n = 1), Tuberculosis (n = 1), Morbi bacterici alii (n = 1), Gonococcal infection (acute) of lower genitourinary tract (n = 1), Neoplasma malignum baseos oris (n = 1), Neoplasma malignum intestini crassi, recto except (n = 1), Neoplasma malignum mammae (n = 1), Neoplasma malignum cervicis uteri (n = 1), Neoplasma benignum systematis respirationis (n = 2), Myoma uteri (n = 3), Neoplasma benignum ovarii (n = 2), Struma nodosa atoxica (n = 1), Morbi glandularum aliarum systematis endocrine (n = 1), Morbi parathyreoideae (n = 1), Morbi glandulae suprarenalis (n = 1), Functio laesa metabolismi proteini plasmatis (n = 1), Persona pathologica asthenica (n = 1), Alcoholismus (n = 1), Perturbationes fortuitae psychogenes accidentals (n = 1), Morbi nervorum et gangliorum periphericorum (n = 1), Alii morbi nervorum cranialium (n = 1), Alii morbi inflammatorii auris (n = 1), Hypertonia benigna essentialis (n = 2), Hypertonia non indicata (n = 1), Angina pectoris (n = 1), Morbus cordis ischaemicus asymptomaticus (n = 1), Ischaemia cerebralis transitoria (n = 1), Varices venarum extremitatum inferiorum (n = 3), Bronchopneumonia (n = 2), Bronchitis chronica (n = 2), Asthma bronchiale (n = 1), Alii morbi tractus respiratorii superioris (n = 1), Laryngitis chronica (n = 1), Rhinitis allergica (n = 1), Ulcus duodeni (n = 1), Appendicitis acuta (n = 3), Hernia abdominalis (n = 4), Gastro-enteritis et colitis non ulcerosa (n = 1), Alii morbi intestinorum et peritonei (n = 1), Cholelithiasis (n = 2), Alii morbi systematis urinarii (n = 2), Morbi organorum genitalium viri (n = 1), Orchitis et epididymitis (n = 1), Morbi mammae, ovarii, tubae, parametrii (n = 1), Alii morbi mammae (n = 1), Morbi ovarii et tubae alii (n = 1), Morbi cervicis uteri alii (n = 1), Aliae complicationes gravidarum (n = 1), Morbi cutis alii (n = 1), Arthritis rheumatoides et morbi similes (n = 1), Osteo-arthritis (arthrosis) et morbi similes (n = 1), Arthritis (n = 1), Rheumatismus alius non articularis (n = 1), Morbi meniscorum et alii morbi cartilagines articuli (n = 5), Alii morbi articulorum (n = 1), Alia symptomata systematis nervosi et organorum sensum (n = 1), Syncope (lipothymia) vasovagalis (n = 1), Symptomata tractus digestionis inferioris (n = 3), Febris incertae causae (n = 1), Nervosimus (n = 1), Cephalalgia (n = 1), Casus mentales pro abortu provocato sive sterilisatione (n = 1), Laceratio et vulnus extremitatis superioris (n = 2), Contusio loci alterius, multiplex sive NUD (n = 1), Contusio sive compressio, cute intacta (n = 2), Investigation of circulatory system (n = 1) and Investigation of genito-urinary system (n = 1). The remaining three hospitalizations had no ICD code recorded.

**Table 1:** Characteristics of the study population.

	Total	HO	NHO	p
N	128	96	32	
Age (years)	59.8 (± 5.5)	61.1 (± 4.9)	55.9 (± 5.4)	**1.0 × 10**^**–6**^
Sex (women)	69 (53.9)	47 (49)	22 (68.8)	0.052
BMI (kg/m^2^)	32.7 (± 3.2)	33.2 (± 3.4)	31.3 (± 1.3)	**0.002**
Waist (cm)	100.5 (± 12.9)	102.6 (± 13.0)	94.2 (± 10.5)	**0.001**
Smoking (yes/no)	22 (17.2)	15 (15.6)	7 (21.9)	0.417
Anti-PC IgM (AU)	113 (98–130)	110 (96–126)	124 (110–137)	**0.008**
Anti-PC IgG1 (AU)	126 (86–203)	110 (77–187)	174 (96–230)	**0.023**
Anti-PC IgG2 (AU)	222 (110–480)	222 (112–455)	261 (85–506)	0.741
SBP (mmHg)	148 (± 17)	150 (± 18)	141 (± 12)	**0.017**
DBP (mmHg)	91 (± 9)	92 (± 9)	88 (± 6)	**0.042**
Total cholesterol (mmol/L)	6.3 (5.7–7.3)	6.2 (5.6–7.4)	6.4 (5.7–7.3)	0.511
HDL-C (mmol/L)	1.2 (± 0.2)	1.2 (± 0.3)	1.2 (± 0.3)	0.724
Triglycerides (mmol/L)	1.5 (1.1–2.9)	1.5 (1.1–2.5)	1.4 (1.0–1.9)	0.240
Fasting blood glucose (mmol/L)	5.2 (4.8–5.7)	5.3 (4.8–5.9)	5.0 (4.6–5.4)	**0.033**
Diabetes mellitus, n (%)	26 (20.3)	24 (25)	2 (6.3)	**0.022**
MetS, n (%)	64 (50)	51 (53.1)	13 (40.6)	0.221
Abdominal obesity, n (%)	47 (36.7)	40 (41.7)	7 (21.9)	**0.044**
High triglycerides, n (%)	53 (41.4)	43 (44.8)	10 (31.3)	0.178
Low HDL-C, n (%)	60 (46.9)	43 (44.8)	17 (53.1)	0.413
Hypertension, n (%)	121 (94.5)	92 (95.8)	29 (90.6)	0.262
Elevated glucose, n (%)	37 (28.9)	31 (32.2)	6 (18.8)	0.143

Bold indicates significance (p≤0.05)

Values are means (± standard deviation), medians (25–75 interquartile range), or numbers (%). Components of the Metabolic syndrome (Abdominal obesity (waist circumference ≥ 88 cm and ≥ 102 cm for women and men, respectively), elevated triglycerides (≥ 1.7 mmol/L), reduced high density lipoprotein cholesterol (< 1.03 mmol/L in males and < 1.29 mmol/L in females), hypertension (systolic blood pressure ≥ 130 mmHg and/or diastolic blood pressure ≥ 85 mmHg, or drug treatment), or elevated fasting glucose (≥ 5.6 mmol/L or glucose-lowering treatment) were defined as stated by Alberti et al.^13^.

*HO* hospitalized obese, *NHO* non-hospitalized obese, *AU* arbitrary units, *Anti-PC* antibodies against phosphorylcholine, *Ig* immunoglobulin, *MetS* metabolic syndrome, *SBP* systolic blood pressure, *DBP* diastolic blood pressure, *HDL* high density lipoprotein cholesterol.

Bold indicates significance (p ≤ 0.05)

### Anti-PC levels and metabolically unhealthy obesity

Each 1 SD increment in anti-PC IgM levels was associated with a lower prevalence of HO when unadjusted, OR 0.53 (CI 95% 0.31–0.90; p = 0.020), and when adjusted for age and sex, OR 0.54 (CI 95% 0.30–0.99; p = 0.049), but the association was attenuated upon further adjustment for waist circumference, SBP, DBP, FBG, and smoking status, OR 0.58 (CI 95% 0.30–1.15; p = 0.120).

Each 1 SD increment in anti-PC IgG1 levels was associated with lower prevalence of HO in unadjusted logistic regressions (OR 0.60; CI 95% 0.39–0.93; p = 0.024), and further adjusted for age and sex (OR 0.58; CI 95% 0.35–0.95; p = 0.029). The association remained significant when waist circumference, SBP, DBP, FBG, and smoking status were entered in the model (OR 0.57; CI 95% 0.33–0.99; p = 0.044), Table 2. Further, sex-specific analyses were carried out, showing association between high anti-PC IgG1 levels and lower prevalence of HO in men, but not in women in the fully adjusted *Model 2* (Table 3). There was a trend for sex-specific associations of anti-PC IgM with HO in women, but this association was attenuated after adjusting for age and sex in *Model 1* (Table 3).

**Table 2:** Associations between anti-PC and risk of being a hospitalized obese subject (HO).

	Anti-PC IgM		Anti-PC IgG1		Anti-PC IgG2	
	OR (CI 95%)	p	OR (CI 95%)	P	OR (CI 95%)	p
**Unadjusted**						
Anti-PC	0.53 (0.31–0.90)	0.020	0.60 (0.39–0.93)	0.024	1.01 (0.68–1.51)	0.947
**Model 1**						
Anti-PC	0.54 (0.30–0.99)	0.049	0.58 (0.35–0.95)	0.029	–	–
Age	1.22 (1.12–1.34)	1.8 × 10^–5^	1.23 (1.12–1.35)	1.4 × 10^–5^	–	–
Sex	0.29 (0.11–0.80)	0.017	0.28 (0.10–0.79)	0.016	–	–
**Model 2**						
Anti-PC	0.58 (0.30–1.15)	0.120	0.57 (0.33–0.98)	0.044	–	–
Age	1.25 (1.13–1.39)	1.9 × 10^–5^	1.27 (1.14–1.42)	1.8 × 10^–5^	–	–
Sex	1.12 (0.24–5.24)	0.889	1.29 (0.26–6.43)	0.760	–	–
Waist circumference	1.09 (1.01–1.18)	0.024	1.10 (1.02–1.19)	0.018	–	–
Systolic blood pressure	1.03 (0.99–1.06)	0.158	1.03 (1.00–1.07)	0.083	–	–
Fasting blood glucose	1.34 (0.66–2.73)	0.416	1.12 (0.55–2.30)	0.753	–	–
Smoking	0.77 (0.21–2.77)	0.685	0.85 (0.23–3.11)	0.804	–	–

Values are odds ratios (OR) with 95% confidence intervals (CI 95%).

*Anti-PC* antibodies against phosphorylcholine, *IgM* immunoglobulin M, *IgG1* immunoglobulin G1, *IgG2* immunoglobulin G2.

**Table 3:** Sex-specific associations between anti-PC and risk of being a hospitalized obese subject (HO).

Anti-PC IgM	Men = 59		Women n = 69	
	OR (CI 95%)	p	OR (CI 95%)	p
**Unadjusted**				
Anti-PC IgM	0.60 (0.25–1.45)	0.257	0.51 (0.26–0.99)	0.049
**Model 1**				
Anti-PC IgM		–	0.46 (0.20–1.02)	0.057
Age		–	1.33 (1.15–1.53)	1.2 × 10^–4^
**Model 2**				
Anti-PC IgM		–		–
Age		–		–
Waist circumference		–		–
Systolic blood pressure		–		–
Fasting blood glucose		–		–
Smoking		–		–
**Anti-PC IgG1**				
Unadjusted				
Anti-PC IgG1	0.28 (0.09–0.85)	0.025	0.76 (0.47–1.23)	0.260
Model 1				
Anti-PC IgG1	0.29 (0.09–0.92)	0.036	0.67 (0.36–1.27)	0.221
Age	1.12 (1.00–1.29)	0.052	1.32 (1.15–1.53)	1.2 × 10^–4^
Model 2				
Anti-PC IgG1	0.26 (0.07–0.98)	0.046	0.66 (0.31–1.41)	0.284
Age	1.11 (0.96–1.30)	0.160	1.38 (1.16–1.63)	2.5 × 10^–4^
Waist circumference	1.05 (0.92–1.20)	0.462	1.12 (1.00–1.25)	0.045
Systolic blood pressure	1.02 (0.96–1.07)	0.564	1.05 (1.00–1.10)	0.068
Fasting blood glucose	3.62 (0.68–19.09)	0.130	0.95 (0.31–2.97)	0.933
Smoking	0.10 (0.00–2.43)	0.159	1.60 (0.23–11.06)	0.635

Values are odds ratios (OR) with 95% confidence intervals (CI 95%).

*MetS* metabolic syndrome, *anti-PC* antibodies against phosphorylcholine, *IgM* immunoglobulin M, *IgG1* immunoglobulin G1.

Anti-PC IgG2 was not associated with HO in the unadjusted analyses (p = 0.9) and was therefore not further analyzed.

### Anti-PC IgG1, the metabolic syndrome and diabetes mellitus

Anti-PC levels were neither associated with prevalence of diabetes mellitus (27 cases; OR 0.78; CI 95% 0.42–1.43; p = 0.414), nor with MetS (OR 0.86; CI 95% 0.52–1.41; p = 0.555). No associations were seen in analyses of associations between anti-PC and each component of MetS, except for elevated glucose levels being associated with anti-PC IgG1 levels (including adjustment for glucose lowering treatment) (Table 4).

**Table 4:** Associations between anti-PC and MetS, including each component of MetS.

	Anti-PC IgM		Anti-PC IgG1	
	OR (CI 95%)	p	OR (CI 95%)	p
MetS	0.78 (0.54–1.12)	0.173	0.86 (0.52–1.42)	0.555
Abdominal obesity	0.83 (0.58–1.19)	0.316	1.03 (0.61–1.72)	0.921
High triglycerides	0.73 (0.51–1.04)	0.084	0.66 (0.39–1.11)	0.116
Low HDL-C	0.98 (0.69–1.39)	0.913	1.34 (0.81–2.23)	0.254
Hypertension	1.29 (0.67–2.53)	0.443	1.54 (0.52–4.52)	0.433
Elevated glucose	0.91 (0.62–1.32)	0.608	0.49 (0.27–0.88)	0.017

Values are odds ratios (OR) with 95% confidence intervals (CI95%). MetS – metabolic syndrome. Components of the Metabolic syndrome (Abdominal obesity (waist circumference ≥ 88 cm and ≥ 102 cm for women and men, respectively), elevated triglycerides (≥ 1.7 mmol/L), reduced high density lipoprotein cholesterol (< 1.03 mmol/L in males and < 1.29 mmol/L in females), hypertension (systolic blood pressure ≥ 130 mmHg and/or diastolic blood pressure ≥ 85 mmHg, or drug treatment), or elevated fasting glucose (≥ 5.6 mmol/L or glucose-lowering treatment) were defined as stated by Alberti et al.^13^.

*Anti-PC* antibodies against phosphorylcholine, *IgM* immunoglobulin M, *IgG1* immunoglobulin G1, *HDL* high density lipoprotein cholesterol, *MetS* metabolic syndrome.

## Discussion

We here report that levels of anti-inflammatory IgG1 and IgM anti-PC are significantly lower among HO than among NHO-individuals. When we controlled for non-modifiable risk factors (age and sex) these associations remained significant. However, when also other factors independently associated with HO (waist circumference, systolic blood pressure, fasting blood glucose and smoking), were included in the model, only IgG1 anti-PC remained significantly associated with protection against HO. In contrast, IgG2 anti-PC was not associated with HO, before or after adjustment for potential confounders. We have not been able to determine IgG3 and IgG4 anti-PC at any significant levels previously and these were therefore not included in the present study^13^. Total IgG anti-PC was not included (since both IgG1 and IgG2 were).

We have previously described metabolically healthy obesity in an observational study, based on a definition of obesity (BMI ≥ 30 kg/m^2^) with no history of hospitalization for somatic disease until mid-life (mean age 56 years) at MDCS baseline^9^. In that study, we observed that metabolically healthy obese individuals had a significantly lower risk of total mortality and incident CVD than metabolically unhealthy individuals. Notably, metabolically healthy obese subjects did not have an increased risk of these end-points when compared to non-obesity controls. Descriptive data suggested that metabolically healthy individuals presented with lower levels of lipids and glucose in blood plasma, alongside with a less sedentary behavior than their HO counterparts^9^.

In a recent study, we investigated subjects from the MDCS-CV further by comparing biomarker associations (lipidomics, metabolomics and proteomics) between metabolically healthy obese and metabolically unhealthy obese where we observed similar descriptive results (unpublished data).

Our findings in relation to anti-PC are in line with previous publications on the role of these antibodies in chronic inflammatory conditions. Most studies have involved IgM anti-PC. We previously reported that IgM anti-PC is associated with protection in atherosclerosis progress among hypertensives^12^, CVD (including both stroke and MI)^14–16^, rheumatic diseases, especially SLE, and other systemic rheumatic diseases, but also RA^6,17,18^ and mortality in chronic kidney disease^19^. In general, these findings have been confirmed by other researchers^20–25^, and extended to other chronic diseases as osteoarthritis^24^. Less is known about other subclasses and isotypes of anti-PC than IgM, even though we determined that IgG1, but not IgG2, shows comparable associations with protection as IgM, in atherosclerosis^13^, SLE^26^, and for mortality in CKD^11^.

Experimental studies support that anti-PC may protect against atherosclerosis, its complications and other types of chronic inflammatory clinical conditions. Anti-PC inhibits pro-inflammatory effects of oxidized and modified lipids exposing PC on endothelial cells (studied on IgG anti-PC)^18^. Another example is immunomodulatory properties with anti-inflammatory effects by IgM anti-PC, promoting polarization of anti-inflammatory T regulatory cells, from healthy donors, atherosclerotic plaques, and also SLE-patients^27^. Mechanisms related to atherosclerosis include IgM anti-PC induced inhibition of uptake of oxLDL by macrophages, which could be an important factor in plaque build-up and development^14^. Since accumulation of dead cells is a major feature of atherosclerosis, IgM anti-PC-induced inhibition of cell death caused by an important inflammatory phospholipid, lysophosphatidylcholine^13^ and increased clearance of dead cells by both IgM^7^ and IgG1 anti-PC^26^ could also play a causative and protective role, inhibiting plaque development.

Animal studies also support an atheroprotective role of anti-PC in atherosclerosis development both using active^28^ and passive^29^ immunization, and also in both SLE^30^, and RA^31^. In line with this is a study where pneumococcal vaccination in a mouse model of atherosclerosis caused increases in different antibodies including anti-PC and a modest but significant decrease of atherosclerosis^32^. We recently demonstrated that brown bears (*Ursus arctos*) which hibernate for 5–6 months during winter, gain weight considerably before hibernation, but despite kidney insufficiency, dyslipidemia and inactivity do not develop atherosclerosis or cardiovascular disease (CVD), have strikingly high levels of IgM and IgG1 anti-PC, thus a potential natural immunization against atherosclerosis^33^.

Obesity is a chronic inflammatory condition, affecting different organs including the adipose tissue. Also the immune system is involved, and immune competent cells are known to infiltrate adipose tissue^34^. Adipose tissue is known to be an endocrine organ where different cell types, including immune competent cells, secrete an array of hormones and cytokines, where the net effect is pro-inflammatory^35^. Interestingly, immunosuppressive, anti-inflammatory T regulatory cells are decreased in obesity. In principle, it is thus possible that low IgM anti-PC could be one factor behind low T regulatory cells in obesity. Further, inflammation can be both a cause and effect of obesity and ensuing metabolic changes^34,36^. An immune-deficient state with low IgM and IgG1 anti-PC could thus potentially promote obesity and related inflammation. Even though IgM anti-PC only remained significant after adjustment for non-modifiable risk factors (age and sex), but not when adjusted for waist circumference, systolic blood pressure, fasting blood glucose and smoking, we consider this finding to be relevant for the difference between NHO and HO. The cross-sectional nature of the study precludes us from drawing any conclusion about causation but still the underlying properties of IgM and IgG1 anti-PC makes causation possible, even plausible, although larger, prospective and experimental studies are needed to prove this.

Human anti-PC’s are often referred to as natural antibodies, based on data from laboratory mice, which we determined as germ-line encoded, with a dominant clone, TI5. In humans, however, we could not detect such a dominant clone, but instead human anti-PC are characterized somatic mutations with Ig-switch and also T cell dependency^7,37^. Humans are born with very low levels of anti-PC, which are not close to their mothers’ levels even after 2 years. This suggests that environmental factors, especially the gut microbiome, could play an important role in development of anti-PC, but that genetic programs also may contribute^38^. Recently, associations between four gut microbiota genera and BMI-predictive plasma metabolites were determined and were found to be possible mediators between gut microbiota and obesity^39^. The possibility that the microbiome is a regulating factor behind low levels of anti-PC in hospitalized obese subjects HO therefore deserves further study.

Other properties of IgM and IgG1 anti-PC as clearance of dead cells, inhibition of cell death caused by inflammatory phospholipids and increased uptake of OxLDL could have a direct effect on complications of obesity, where atherosclerosis and CVD are of major importance.

Individuals from Kitava, New Guinea, were studied in the early 1990s, and were found to have a very favorable metabolic profile, where obesity, metabolic disorders, hypertension and type 2 diabetes were virtually absent. Undoubtedly, one explanation could be differences in lifestyle, diet and exercise. We also reported that levels of IgG and IgM anti-PC are significantly lower among Swedish sex- and age-matched controls than Kitavans and based on these findings we proposed a development of the Hygiene/Old Friends hypothesis. This states that a lack of exposure to PC-bearing microorganisms such as nematodes, parasites, and also some bacteria (including *Treponema*) results in low levels of anti-PC and ensuing increased risk of atherosclerosis, CVD, and other chronic inflammation. Here, we could add obesity and metabolic alterations, based on the present data^6,40–42^.

Another finding is that IgG1 and IgG2 anti-PC differ completely in relation to HO and NHO: while IgG1 was a significant marker of protection even after controlling for several other potential confounders, IgG2 was not. This finding is in line with our previous studies on these antibodies, where IgG1, but not IgG2 anti-PC, was associated with protection in atherosclerosis progress^13^, SLE^11^ and mortality in CKD^6^.

PC can also be presented as p-nitrophenyl phosphorylcholine (NPPC)^13^ and anti-PC may be divided into group I (IgM and IgG1) and group II (IgG2)^13^. Group I anti-PC recognizes both forms of PC but group II antibodies only recognize NPCC, where the phenyl-ring attached to PC is involved in the antigenicity. IgG2 anti-PC is directed against capsulated bacteria, recognizes carbohydrate antigens, and has bactericidal properties^13,43,44^. It is thus likely that the most protective immune response to PC is not derived from PC on carbohydrate structures of capsulated bacteria.

Further, the present finding that IgG1 anti-PC was significantly protective against hospitalization among obese men but not women, also after controlling for potential confounders, is in line with our previous findings, where associations among men are more prominent^6^. IgM anti-PC was a significant protective marker among women (not controlled for confounders), why it is difficult to draw conclusions about sex differences in this context.

There are limitations to this study. One is that it is relatively small, and it is therefore difficult to determine associations and also control for confounders due to lack of power. Further, the cross-sectional nature of the study precludes any conclusions about causation. It would have been of interest to study obesity in general as compared to matched controls, which is not included herein.

In conclusion, we here demonstrate that anti-inflammatory IgM and IgG1, but not IgG2 anti-PC, are inversely associated with higher risk of being HO, also after controlling for sex and age. However, only IgG1 anti-PC remained significant when also other potential confounders were controlled for. We still think that also IgM is of interest due to its other properties, especially anti-inflammatory, which could be causally related to these factors. In general, IgM and especially IgG1 could be protective for obesity complications, a mechanism that could have implications for prediction of risk, but also for prevention through immunization with PC.

## Abbreviations

Anti-PC: Antibodies against phosphorylcholine
CVD: Cardiovascular disease
HO: Hospitalized obese
MDCS-CV: Malmö Diet Cancer Study-Cardiovascular cohort
NHO: Non-hospitalized obese
MetS: Metabolic syndrome
OxLDL: Oxidization of low-density lipoproteins
